# The Association between Intelligence and Telomere Length: A Longitudinal Population Based Study

**DOI:** 10.1371/journal.pone.0049356

**Published:** 2012-11-14

**Authors:** Eva M. Kingma, Peter de Jonge, Pim van der Harst, Johan Ormel, Judith G. M. Rosmalen

**Affiliations:** 1 Interdisciplinary Center Psychopathology and Emotion Regulation (ICPE), University of Groningen, University Medical Center Groningen, Groningen, The Netherlands; 2 Department of Cardiology, University of Groningen, University Medical Center Groningen, Groningen, The Netherlands; Federal University of Rio de Janeiro, Brazil

## Abstract

Low intelligence has been associated with poor health and mortality, but underlying mechanisms remain obscure. We hypothesized that low intelligence is associated with accelerated biological ageing as reflected by telomere length; we suggested potential mediation of this association by unhealthy behaviors and low socioeconomic position. The study was performed in a longitudinal population-based cohort study of 895 participants (46.8% males). Intelligence was measured with the Generalized Aptitude-Test Battery at mean age 52.8 years (33–79 years, SD = 11.3). Leukocyte telomere length was measured by PCR. Lifestyle and socioeconomic factors were assessed using written self-report measures. Linear regression analyses, adjusted for age, sex, and telomere length measured at the first assessment wave (T1), showed that low intelligence was associated with shorter leukocyte telomere length at approximately 2 years follow-up (beta = .081, t = 2.160, p = .031). Nearly 40% of this association was explained by an unhealthy lifestyle, while low socioeconomic position did not add any significant mediation. Low intelligence may be a risk factor for accelerated biological ageing, thereby providing an explanation for its association with poor health and mortality.

## Introduction

Low intelligence or low cognitive ability has been associated with poor health, including aging-related diseases, and mortality [1]–[6]. Two non-exclusive pathways explaining the link between intelligence and health are commonly suggested. First, low intelligence individuals tend to find themselves in more adverse environments including psychological stress [6]–[9]. Second, low intelligence individuals engage themselves in more unhealthy behaviors [10], [11]. As both stress [12], [13] and unhealthy behaviors [14] are associated with morbidity and mortality, they are considered as possible mediators in the association between intelligence and health. The question arises as to which biological mechanisms mediate the association between intelligence and health. One possible mechanism might be the shortening of telomeres. Telomere length has been linked with a spectrum of ageing-related diseases and mortality [15]–[17]. Telomere length and attrition is believed to capture biological ageing above and beyond chronological age, such that shorter telomeres represent increased biological senescence [18]. Therefore, telomere length is often considered a useful measure of biological age. Furthermore, telomere length is influenced by psychological stress [19]–[22] and unhealthy behaviors [23]–[25]. Given the associations of these two factors with low intelligence, low intelligence might be associated with shorter telomere length.

Previous studies on telomere length and cognitive ability presented mixed findings [26]–[28]. However, variations in findings may be due to differences in cognitive tests used, the use of cross-sectional data on telomere length, and the use of case-specific samples with regard to age [27], [29] and sex [28]. In addition, these former studies focused mainly on shortened telomere length as a potential risk factor for cognitive decline, but ignored the possibility of reverse causality, that is the effect of cognitive ability or intelligence on telomere length. One recent prospective study examined the effect of general cognitive ability at age 11 and telomere length at age 70, but found no significant effect [29]. However, results may not be applicable to other age ranges, as prospective effects of intelligence on telomere length may change across the lifespan. Therefore, reversed causality remains unclear and needs to be addressed in a longitudinal design including a broad age-range. Furthermore, reverse causality would also imply alternative mediating mechanisms, namely those that have been shown to play a role in the association between intelligence and morbidity. While candidates such as socioeconomic and lifestyle factors were treated as confounders in the association between telomere length and cognitive ability [27], [28], their potential role as mediators was ignored.

Our aim was to study the prospective association between intelligence and telomere length in a general population cohort of males and females with a broad age-range. We hypothesized that low intelligence predicts shorter telomere length. Moreover, we hypothesized that part of the association between low intelligence and shorter telomere length is explained by low socioeconomic position and unhealthy behaviors.

## Methods

### Sample

Our study has been performed in a cohort derived from Prevention of REnal and Vascular ENd stage Disease (PREVEND), a population cohort study investigating microalbuminuria as a risk factor for renal and cardiovascular disease. The recruitment of participants has been extensively described elsewhere [30]. All inhabitants of the city of Groningen between the ages of 28 and 75 yrs (85,421 subjects) were asked to send in a morning urine sample and to fill out a short questionnaire on demographics and cardiovascular history. A total of 40,856 subjects (47.8%) responded. After exclusion of subjects with insulin dependent diabetes mellitus and pregnant women, all subjects with an elevated urinary albumin concentration of greater than 10 mg/L (N = 7768) together with a randomly selected control group with a urinary albumin concentration of <10 mg/L (N = 3395) were invited for further investigations (total N = 11,163). Finally, 8592 subjects completed the total screening program in 1997–1998 forming the PREVEND baseline population (T1). Because the PREVEND study population was enriched for albuminuria, this oversampling for albuminuria was counterbalanced in the current sub study. Albuminuria-negative participants and a random sample of albuminuria-positive participants were combined so that a population-representative ratio of albuminuria-positive participants was achieved. In 2001–2003, during the second screening of PREVEND, research assistants handed over invitations to 2554 subjects to participate in the current sub study for which additional psychiatric and psychosocial data were collected. Of these 2554 subjects, 1094 (43%) completed the additional measurements, forming the baseline population cohort of the current study (T2). The participants who declined to participate in the additional data collection did not significantly differ from those who did participate concerning sex or age. Follow-up measurements in the 2003–2004 wave (T3) were completed by a total of 976 participants (89% of the cohort with additional psychiatric and psychosocial data), forming the cohort for the current study. Dropout participants (n = 118) were older (mean age ± SD 56.6±11.9 vs. 52.7±11.2 years, t = 3.66, p<0.01) and more often female (66 vs. 52%, χ^2^ = 8.13, p<0.01). The study was approved by the local medical ethics committee. All subjects provided written informed consent to participate in the study.

### Assessment of intelligence

General intelligence was measured at T2 using the computerized version of the General Aptitude-Test Battery (GATB) version B 1002-B [31]. The GATB consists of a combination of tests that measure nine aptitudes. The aptitude general intelligence is measured by three tests: 1) a dimensional space test, 2) a vocabulary test, and 3) an arithmetic reasoning test. All participants performed the intelligence test in groups of at most nine persons. Before the start of each test, a test assistant provided test-instructions and provided computer help when necessary. Tests were not started before the test assistant had ensured that the participants had successfully completed the practice sessions. To construct a general intelligence score, we summed standardized scores on each of the three subtests. From the 1094 participants, a total of 1061 completed the GATB.

### Assessment of telomere length

Telomere length measurements were performed in a blinded fashion. To avoid any impact of variation in DNA extraction method on telomere length measurement, all samples analyzed in the current study were extracted uniformly using the same DNA extraction kit (QIamp, Qiagen, Venlo, the Netherlands) from frozen full-blood samples. Telomere length was measured in leukocytes using a novel multiplex monochrome real time quantitative polymerase chain reaction technique [32]. This technique allows carrying out the telomere specific amplification and the reference gene amplification in a single reaction well with quantification measurements at different temperatures [32]. All samples were measured in triplicate and the average of the three runs was used to provide the mean relative measure of telomere length for each individual. The mean telomere repeat sequence copy number (T) was compared to a reference single copy gene copy number (S) in each sample. For quality control samples were checked for concordance between triplicate values. When the coefficient of variation (CV) was ≥10% within the triplicate these were re-run. If the CV remained ≥10% the sample was omitted from the statistical analyses. The intra-assay CV was 2% (T), 1.9% (S) and 4.5% (T/S ratio). Reproducibility data was obtained for 216 subjects from PREVEND and good agreement between T/S ratios was observed (R^2^ = 0.99, P<0.0001, inter-run CV 3.9%). Of the 1094 participants included in the population cohort at T2, 1019 had telomere length measurements at T1 and 982 participants at T3, 6 years apart.

### Assessment of lifestyle factors

Lifestyle factors were measured at T2. Body mass index (BMI) was calculated as the ratio between weight and the square of height (kg/m2). Smoking and exercise frequency were assessed by self-report questionnaires. Smoking was categorized as non-smoker, 1–5, 6–10, 11–15, 16–20 or more than 20 cigarettes per day. Frequency of exercise was categorized as not/hardly, once per week, twice or more per week. We previously showed that intelligence is negatively associated with BMI, smoking, and positively associated with exercising [10]. Since there was no linear association between intelligence and alcohol consumption in our former research [10], we did not include it as a potential mediator in the analyses.

### Assessment of socioeconomic position

Socioeconomic position was used as a proxy for adverse environments. Information on income, educational level, and work situation was retrieved from questionnaires that were administered at T1. Income was measured through the gross monthly household income (<1200, 1200–1799, 1800–2199, 2200–2799, 2800–3799, 3800–5800, or >5800 guilders) divided by the square root of the number of people living in the household [33]. One Euro equals about 2.2 guilders. The variable educational level was made up of the following categories: not applicable, low, middle, or high educational level. ‘Low educational level’ was defined as: lower secondary education or less, ‘middle educational level’ as: higher secondary education, and ‘high educational level as: tertiary or further education. Participants with educational category ‘not applicable’ were not dropped from the study. Work situation was categorized in the following three categories: employed (i.e., currently having a job), willingly unemployed (i.e., housekeeping or retired) or unwillingly unemployed (i.e., job seeker or unable to work). We previously showed that intelligence is positively associated with educational level, a more favorable work situation, and higher income [10].

### Statistical analyses

We performed all analyses using the Statistical Package for the Social Sciences version 18.0 (SPSS Inc, Chicago, IL, USA). Leukocyte telomere length was not normally distributed and was therefore linear transformed. We performed univariable linear regression analyses between all potential predictors (intelligence, age, sex, telomere length at T1, lifestyle factors, and socioeconomic) and telomere length at T3. In addition, we subtracted telomere length at T3 from telomere length at T1 and dichotomized outcome scores in ‘0’ (n = 454, 50.7%) for all results below zero (indicating telomere length shortening) and ‘1’ (n = 441, 49.3%) for all results higher than zero (indicating telomere length elongation). Both groups were compared on mean intelligence scores. Since previous research on telomere length and cognition showed differences between males and females [29], we tested for potential interaction by sex and intelligence in predicting telomere length, but this was not statistically significant (beta = .001, t = .020, p = .984). Therefore, all further analyses were performed using the whole group. Multivariate linear regression analyses were performed to test whether intelligence was associated with leukocyte telomere length at T3, while adjusting for age, sex, and telomere length at T1. We used a bootstrapping procedure developed by Preacher and Hayes [34] to test the extent to which lifestyle factors mediated the association between intelligence and telomere length at T3. Then we tested to what extent lifestyle plus socioeconomic factors mediated that association. Bootstrapping is a method that involves repeatedly sampling from the original data set and estimating the indirect effect in each resampled data set [34]. By repeating this process a thousand times, an empirical approximation of the sampling distribution is built [34]. Then it is used to construct 95% confidence intervals for the total indirect effect of intelligence on telomere length at T3, via lifestyle factors and socioeconomic factors.

## Results

In total, 895 participants completed measurements of intelligence and had telomere length measurements at T1 and T3. Sample characteristics are depicted in table 1.

**Table 1:** Sample characteristics.

Measure	(%)	Mean (SD)
**Age** 1		52.8 (11.3)
**Female sex**	53.2	
**Lifestyle factors**		
**BMI** 2		26.4 (3.9)
**Smoking**		
0 cigarettes/day	76.9	
1–5 cigarettes/day	4.5	
6–10 cigarettes/day	3.9	
11–15 cigarettes/day	6.5	
16–20 cigarettes/day	4.7	
>20 cigarettes/day	3.4	
**Exercise frequency**		
No exercise	50.3	
Once/week	28.0	
Twice or more/week	21.2	
**Socio-economic factors**		
**Educational level**		
Not applicable	4.2	
Low	24.1	
Middle	25.4	
High	39.2	
**Work situation**		
Employed	57.3	
Willingly unemployed	25.7	
Unwillingly unemployed	8.5	
**Income** 3		2521.1 (951.3)
**Morbidity**		
Diabetes^4^	2.3	
Coronary heart disease^5^	4.6	

1 Age in years at T2.

2 Body mass index in kg/m^2^.

3 Household income (in guilders).

4 Defined as the use of antidiabetic treatment according to self-report or pharmacy data.

5 Defined as self-report of CHD upon inclusion in the study and/or confirmed occurrence of CHD between inclusion and date of psychiatric interview.

### Univariable associations

**Figure 1:**
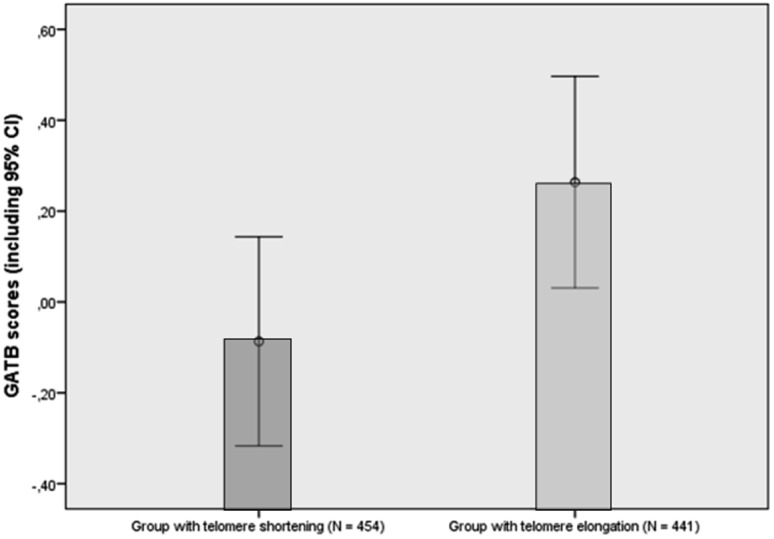
Mean intelligence scores of participants with telomere length shortening and telomere length elongation. ifference in scores on the GATB: F = .049, p = .036.

Depicted in table 2 are the univariable associations between the potential predictors (intelligence, age, sex, telomere length at T1, lifestyle factors, and socioeconomic factors) and telomere length at T3. Data were explored for linear associations. Intelligence, exercise frequency, educational level, and work situation were significantly positively associated with telomere length at T3, while age and BMI were significantly negatively associated with telomere length at T3. In contrast, sex, smoking, and income were not significantly associated with telomere length at T3. In addition, the association between intelligence and telomere length at T1 approached significance (p = .06). As a post hoc analysis, we looked at the change in telomere length by subtracting telomere length at T3 from telomere length at T1. Figure 1 reveals that mean GATB scores of the group that showed telomere length shortening (n = 454, 50.7%) were significantly lower than mean GATB scores of the group that showed telomere length elongation (n = 441, 49.3%) (F = .049, p = .036).

**Table 2:** Univariable associations between predictors and telomere length at T3.

	beta	t	p
**Intelligence**	.111	3.319	.001
**Age** 1	−.138	−4.359	<.001
**Female sex**	.056	1.755	.080
**Telomere length at T1**	.200	6.177	<.001
**Lifestyle factors**			
BMI^2^	−.116	−3.666	<.001
Smoking^3^	.008	−1.269	.205
Exercise frequency^4^	.127	3.989	<.001
**Socio-economic factors**			
Educational level^5^	.078	2.346	.019
Work situation^6^	.104	3.125	.002
Income^7^	.016	.446	.656

1 Age in years at T2.

2 Body mass index in kg/m^2^.

3 Smoking in number of cigarettes/day with “0” = 0, “1” = 1–5, “2” = 6–10, “3” = 11–15, “4” = 16–20”, “5” = >20.

4 Frequency of exercise with “0” = no exercise, “1” = once/week, “2” = twice or more/week.

5 Educational level with “1” = none, “2” = low, “3” =  middle, “4” = high.

6 Work situation with “1” = unwillingly unemployed, “2” = willingly unemployed, “3” = employed.

7 Household income divided by the square root of number of people living with this income.

### Multivariable associations between intelligence and telomere length

The multivariable associations between intelligence and telomere length are presented in table 3. Low intelligence significantly predicted shorter telomere length at T3, with a standardized beta somewhat higher than that of age. As a sensitivity analysis, we examined the association between intelligence and telomere length at T3 limited on the group younger than 70 years. No substantial change in results was found (beta = .095, t = 2.499, p<.013).

**Table 3:** Multivariable associations between intelligence and telomere length at T3.

	beta (R^2^)	t	p
	(.047)		
**Age** 1	−.098	−2.975	.003
**Female sex**	.025	.784	.433
**Telomere length at T1**	.180	5.459	<.001
	(.050)		
**Intelligence**	.081	2.160	.031
**Age** 1	−.060	−1.617	.106
**Female sex**	.043	1.279	.043
**Telomere length at T1**	.180	5.402	<.001
	(.063)		
**Intelligence**	.049	1.299	.194
**Age** 1	−.049	−1.289	.198
**Female sex**	.036	1.053	.293
**Telomere length at T1**	.173	5.190	<.001
**Lifestyle factors**			
BMI^2^	−.060	−1.760	.079
Smoking^3^	−.041	−1.209	.227
Exercise frequency^4^	.107	3.208	.001
	(.072)		
**Intelligence**	.002	.047	.962
**Age** 1	−.142	−2.935	.003
**Female sex**	.022	.526	.599
**Telomere length at T1**	.163	4.103	<.001
**Lifestyle factors**			
BMI^2^	−.088	−2.126	.034
Smoking^3^	−.048	−1.169	.243
Exercise frequency^4^	.084	2.078	.038
**Socio-economic factors**			
Educational level^5^	−.017	−.335	.738
Work situation^6^	−.018	−.404	.686
Income^7^	.062	1.407	.160

1 Age in years at T2.

2 Body mass index in kg/m^2^.

3 Smoking in number of cigarettes/day with “0” = 0, “1” = 1–5, “2” = 6–10, “3” = 11–15, “4” = 16–20”, “5” = >20.

4 Frequency of exercise with “0” = no exercise, “1” = once/week, “2” = twice or more/week.

5 Educational level with “1” = none, “2” = low, “3” =  middle, “4” = high.

6 Work situation with “1” = unwillingly unemployed, “2” = willingly unemployed, “3” = employed.

7 Household income divided by the square root of number of people living with this income.

When lifestyle factors were included in the model, the beta of intelligence fell from .081 (t = 2.160, p = .031) to .049 (t = 1.299, p = .194). Bootstrapping revealed that the total indirect effect of intelligence on telomere length at T3, via lifestyle factors, was statistically significant, with a 95% confidence interval ranging from .014 to .073. Therefore, 39.5% of the association between intelligence and telomere length at T3 was statistically mediated by lifestyle factors. Additionally, we included both lifestyle and socioeconomic factors in the model and then the beta of intelligence fell from .081 (t = 2.160, p<.05) to .002 (t = .047, p>.962). Bootstrapping revealed that the total indirect effect of intelligence on telomere length at T3, via lifestyle and socioeconomic factors, was not statistically significant, with a 95% confidence interval ranging from −.021 to .101.

As a post hoc analysis, we tested the mediating effect of the individual life style factors. When BMI was included in the model, the beta of intelligence fell from .081 (t = 2.160, p<.031) to .071 (t = 1.896, p>.058). Bootstrapping revealed that this indicated a significant indirect effect, with a 95% confidence interval of .001 to .024. When smoking was included into the model, the beta of intelligence fell from .081 (t = 2.160, p<.05) to .067 (t = 1.772, p>.077). Bootstrapping revealed that this indirect effect was not significant. Lastly, when frequency of exercise was included into the model, the beta of intelligence fell from .081 (t = 2.160, p<.031) to .065 (t = 1.755, p>.080). Bootstrapping revealed that this indicated a significant indirect effect, with a 95% confidence interval of .001 to .025. As another post-hoc analysis, we specifically looked at the role of educational level. Educational levels and intelligence were significantly correlated (Pearson's r = .56, p<.01). When educational level was entered in the model (including age, sex, and telomere length at T1) instead of intelligence, educational level was not significantly associated with telomere length at T3 (beta = .038, t = 1.069, p = .286).

## Discussion

This paper reports three key findings. First, intelligence was positively associated with telomere length at follow-up. Second, nearly 40% of this association was explained by an unhealthy lifestyle, but low socioeconomic position did not add any significant mediation. Third, high BMI and low frequency of exercise were independent predictors of shorter leukocyte telomere length over time.

One of the major strengths of this study is that we used a large longitudinal population based cohort including repeated measures of telomere length, while many other studies on telomere length attrition are based on cross-sectional data from specific patient groups. This enabled us to study intelligence as a potential predictor of shorter telomere length in a longitudinal design. In addition, we studied the mediating effect of several lifestyle and socioeconomic factors. Despite the strengths of our findings outlined above, there are also limitations that should be considered when interpreting our results. The first measure of telomere length and intelligence were not assessed at the same point in time. Since intelligence could be considered as a stable factor [35] and the time span between both assessment waves is relatively small, it is unlikely that this has biased our results. Still, it might be that performance on the GATB is not stable across the lifespan. However, after repeating the analyses on participants younger than 70 years, the association between intelligence and telomere length did not change significantly. Therefore, it is unlikely that our findings were the result of cognitive decline. Validations of the GATB are mostly based on the value of this test to predict school and job performance. A drawback of this test is that two (vocabulary and arithmetic reasoning test) of the three subtests to measure intelligence may depend largely on education. Nevertheless, although intelligence and educational level are well correlated, they are not interchangeable and seem to play different roles in relation to telomere length. Indeed, post-hoc analysis showed that when educational level is entered in the model (including age, sex, and telomere length at T1) instead of intelligence, educational level was not significantly associated with telomere length at T3. ‘Psychological stress’ was hypothesized as a potential mediator, but crude socioeconomic indicators for adverse environments were used. Adverse environments, such as financial strain or unemployment, are recognized markers of psychological stress, but may not capture the entire concept. Cross-sectional data of lifestyle factors were used, although these may be subject to change over time.

To our knowledge, this is the first prospective study on the association between intelligence and telomere length in a large population-based cohort, including a broad age-range and longitudinal data on telomere length. Although we cannot draw any conclusions regarding direct causality, our results suggest that lower intelligence result in shorter telomere length. Intelligence positively predicted leukocyte telomere length at follow-up, independent of both predictors age and telomere length at T1. In multivariable analysis, intelligence was a stronger predictor of telomere length than chronological age. Overall, former studies lack referral to reverse causality, where lower intelligence predicts shorter telomere length at follow-up. One recently published prospective study found no significant association between general cognitive ability at age 11 and telomere length at age 70 [29], but did find a cross-sectional association between general cognitive ability and telomere length at age 70, only in females [29]. Our interaction analyses did not indicate a statistically significant interaction between intelligence and sex in predicting telomere length; therefore, further analyses were performed in the whole group. It may be that the males in the previous study of only 70-year-old participants had longer telomeres than their contemporaries who died at a younger age [29]. Although we cannot draw any conclusions regarding direct causality, our results suggest that lower intelligence result in shorter telomere length.

An important part of the association between intelligence and telomere length was explained statistically by lifestyle factors. More specific, higher BMI and physical inactivity were independent predictors of telomere length. Previous studies, although particularly in women, also found an inverse relation between BMI and telomere length [23], [36], [37], whereas a study that focused only on men [38] found no association between BMI and telomere length. Moreover, a positive association between exercise and telomeres has been described in former research [24], [25]. Different mechanisms have been suggested to explain the association between BMI and exercise frequency and telomere length. High BMI has been associated with inflammation [39], a process marked by increased white blood cell turnover and consequently lower leukocyte telomere length [23]. Furthermore, telomerase activity, a reverse transcriptase enzyme that adds telomeric DNA to shortened telomeres [40], has been suggested to be augmented after exercise [25]. Unfortunately, we did not have data on telomerase activity. In addition, both lifestyle and socioeconomic position attenuated the association between intelligence and telomere length at follow up, but did not add any significant mediation. Although we did observe correlations between the individual socioeconomic factors and telomere length, when intelligence was added to the model, the association between socioeconomic factors and telomere length did not hold. The previous prospective study [29] found no association between both social class and lifestyle and telomere length. However, the relatively homogeneous cohort, at least concerning social class, may not be varied enough to detect significant associations. Former studies focusing on socioeconomic inequalities in health show that intelligence indeed explains some of the socioeconomic gradient in health [41], [42], including mortality, which in itself is linked to shorter telomere length [17]. It is possible that the association between socioeconomic status and telomere length was removed by including age in the model, since age is a strong correlate of socioeconomic factors. We found no significant association between educational level and telomere length. Previous studies showed positive associations as well as no associations [43]. It could be speculated that associations between educational level and telomere length reflect those between intelligence and telomere length. The mixed results could be caused by differences between cohorts in the strength of the relation between intelligence and educational level, given the fact that this relation is influenced by sociocultural factors. As an additional remark, it should be kept in mind that the explained variances of the models tested are small (5–7%).

In conclusion, low intelligence may be a risk factor of accelerated biological ageing and this could explain part of the link between low intelligence and poor health and mortality. A substantial part of the association between intelligence and telomere length over time was explained by an unhealthy lifestyle.
